# Computational study on new natural compound inhibitors of Traf2 and Nck-interacting kinase (TNIK)

**DOI:** 10.18632/aging.204349

**Published:** 2022-10-25

**Authors:** Lushun Ma, Rui Li, Zhiwei Yao, Bo Wang, Yong Liu, Chunxiang Liu, Heng Wang, Shuxian Chen, Daqing Sun

**Affiliations:** 1 Department of Paediatric Surgery, Tianjin Medical University General Hospital, Tianjin, China; 2 Department of Gastrointestinal Surgery/Pediatric Surgery, Renmin Hospital, Hubei University of Medicine, Shiyan, Hubei, China

**Keywords:** colorectal cancer, TNIK, inhibitor, virtual screening, natural small molecules

## Abstract

Traf2 and Nck-interacting kinase (TNIK) is the downstream molecule of Wnt/β-catenin signal pathway. As the activation kinase of β-catenin/T-cell factor 4 transcription complex, it can fully activate Wnt signalling and promote the growth and invasion of tumor cells. We conducted computer-assisted virtual screening and a series of analyses to find potential inhibitors of TNIK. First, LibDock was used for molecular docking of natural small molecules. Then, ADME (Adsorption, Distribution, Metabolism and Excretion) analysis and toxicity prediction were performed on the top 80 small molecules which have higher scores. Additionally, in order to further determine the affinity and binding mechanism of TNIK-ligands, we analyzed the pharmacophores and used CDOCKER for more accurate molecular docking. Last but not least, molecular, dynamics simulation was used to evaluate the stability of receptor-ligand complexes in natural environment. The results showed that natural small molecules (ZINC000040976869 and ZINC000008214460) had high affinity and low interaction energy with TNIK. They were predicted to have excellent pharmacological properties, such as high plasma protein binding capacity and water solubility, no hepatotoxicity, no blood-brain barrier permeability and tolerant with cytochrome P450 2D6 (CYP2D6). In addition, they have less rodent carcinogenicity, AMES mutagenicity, and developmental toxicity potential. Molecular dynamics simulations showed that the two compounds could achieve the stability of potential energy and Root-Mean-Square Deviation (RMSD) at different time nodes. This study proves that ZINC000040976869 and ZINC000008214460 are ideal lead compounds with inhibition targeting to TNIK. These compounds provide valuable ideas and information for the development of new colorectal cancer targeting drugs.

## INTRODUCTION

Colorectal cancer (CRC) is a worldwide disease that seriously harms human health. According to the latest global cancer statistics in 2020, CRC is the third most common cancer in the world, and it has caused more than 900,000 deaths. Its mortality rate has reached the second highest among cancers [1]. Not only that, the incidence of CRC continues to rise along with people's tendency towards red meat diet, reduced physical activity and increased body mass index [2–4]. Surgery, as the main treatment, has developed rapidly in recent years, but it is only effective for CRC patients without lymph nodes and distant metastasis [5]. Therapeutic antibodies against epidermal growth factor receptor (EGFR) and vascular endothelial growth factor (VEGF) combined with chemotherapy, as an important treatment for metastatic or recurrent CRC, have been widely used in clinic in recent years, but they have not significantly increased the 5-year survival rate of patients with advanced CRC [6]. Therefore, it is a very hot topic to find related therapeutic targets and to study new targeted drugs for CRC.

The alteration of Wnt signaling pathway plays a crucial role in the occurrence and development of CRC by affecting the stemness, metastasis and tissue repair of tumor cells [7, 8]. About 80% of colorectal cancers are thought to be related to the activation of components in Wnt signaling pathway caused by mutation of adenomatous polyposis coli (APC) gene [9]. The mutation of APC gene may account for the degradation failure of β-catenin located downstream of Wnt signaling pathway [10]. The β-catenin protein is transported into the nucleus to further activate Wnt signaling. The accumulation of β-catenin in the nucleus activates T-cell factor 4 (TCF4) and forms a complex with it. TCF4 is a member of TCF/LEF transcription factor family, which is an essential factor leading to CRC [9, 11]. Due to the genetic inactivation of APC, only the molecules located downstream of APC are considered as effective targets in Wnt signaling pathway.

Traf2 and NCK-interacting kinase (TNIK) is a member of STE20 serine/threonine protein kinase family, which has an N-terminal activation domain and can specifically activate the c-Jun N-terminal kinase pathways like many germinal center kinases [9, 12]. TNIK is the downstream signal protein and nuclear coactivator of Wnt signaling pathway, which is mainly located in the nucleus. In addition, it is a component of the TCF4 and β-catenin transcriptional complex in CRC cells [13, 14]. When combined with TNIK, the conserved serine 154 residue on TCF4 protein can be phosphorylated by it [14, 15]. This effect is indispensable for the complete activation of Wnt signaling pathway and the growth of CRC cells. Therefore, TNIK is a feasible drug therapy target for rectal cancer caused by abnormal Wnt signaling pathway. NCB-0846 is a TNIK inhibitor, which has been widely studied. It can combine with the ATP activity binding pocket of TNIK and completely inhibit Wnt signal transduction. A study showed that oral administration of NCB-0846 significantly inhibited the growth of tumor transplanted into immunocompromised mice [7]. However, due to its poor pharmacokinetics and low activity, it has not been widely used in clinic [16]. Based on the above reasons, we need to explore safer and more effective TNIK inhibitors for the treatment of CRC.

Natural products and their derivatives have brought new horizons to drug research and development in recent years because of their potential biological functions and unique molecular structure, and have become an important source for the pharmaceutical industry to develop new drugs [17, 18]. This study intends to identify potential novel TNIK inhibitors based on the ZINC database using molecular docking, pharmacological analysis, and molecular dynamics simulations [19, 20].

## RESULTS

### Virtual screening of potential inhibitors of TNIK

The ligand-binding pocket of TNIK is an essential regulatory site for its activity. NCB-0846 can bind to this pocket region to inhibit the function of TNIK in normal environment. Therefore, we choose this pocket as the reference area. ZINC15 database is a free commercial database provided by Irwin and Shoichet Laboratories in the Department of Pharmaceutical Chemistry at the UCSF. We obtained a total of 14962 natural, named and purchasable small molecules from the ZINC15 database. NCB-0846 was used as a reference compound to evaluate the binding capacity of the candidate small molecules. After the LibDock module calculation of DS4.5, it was found that 2,403 compounds could stably bind to TNIK. Among them, 1246 compounds scored higher than NCB-0846 (LibDock score: 111.272, ranking: 1247). The top 80 compounds are listed in Table 1.

**Table 1:** Top 80 ranked compounds with higher Libdock scores than NCB-0846.

**Number**	**Compounds**	**Libdock score**	**Number**	**Compounds**	**Libdock score**
1	ZINC000004099068	174.688	41	ZINC000004096890	145.81
2	ZINC000085545908	170.906	42	ZINC000014233122	145.711
3	ZINC000049784088	167.506	43	ZINC000056897657	145.657
4	ZINC000008552069	166.995	44	ZINC000049878225	145.657
5	ZINC000014951658	164.759	45	ZINC000085544839	145.653
6	ZINC000013513540	161.868	46	ZINC000004097774	145.195
7	ZINC000038143594	161.118	47	ZINC000004099069	145.064
8	ZINC000014952116	156.409	48	ZINC000014686472	144.996
9	ZINC000040976869	155.254	49	ZINC000100288506	144.993
10	ZINC000085826837	154.936	50	ZINC000028467879	144.92
11	ZINC000004096878	154.792	51	ZINC000038143593	144.839
12	ZINC000004096059	154.699	52	ZINC000004095530	144.775
13	ZINC000004096894	154.13	53	ZINC000002528509	144.754
14	ZINC000004096684	153.981	54	ZINC000011536135	144.749
15	ZINC000085541163	153.314	55	ZINC000100590636	144.687
16	ZINC000008551213	152.682	56	ZINC000002528510	144.549
17	ZINC000004096889	152.514	57	ZINC000014712793	144.529
18	ZINC000009212427	152.072	58	ZINC000004228265	144.385
19	ZINC000095562852	152.022	59	ZINC000015721425	144.178
20	ZINC000004228266	151.584	60	ZINC000004096653	144.123
21	ZINC000004096888	150.69	61	ZINC000034944433	143.736
22	ZINC000004096877	150.475	62	ZINC000040165309	143.681
23	ZINC000002526388	150.222	63	ZINC000100277550	143.609
24	ZINC000042805482	149.736	64	ZINC000004228237	143.462
25	ZINC000150338786	149.668	65	ZINC000004096892	143.377
26	ZINC000004228235	149.429	66	ZINC000002566164	143.05
27	ZINC000004228238	149.163	67	ZINC000026671872	142.946
28	ZINC000002005305	148.915	68	ZINC000004016719	142.821
29	ZINC000004096893	148.858	69	ZINC000003927222	142.475
30	ZINC000014811803	148.685	70	ZINC000049872065	142.276
31	ZINC000004228247	148.355	71	ZINC000013451339	142.215
32	ZINC000009212425	148.314	72	ZINC000017044428	142.204
33	ZINC000004096891	148.037	73	ZINC000001530788	142.151
34	ZINC000014951634	148.003	74	ZINC000002033589	141.959
35	ZINC000014946303	147.613	75	ZINC000004096895	141.92
36	ZINC000002526389	147.18	76	ZINC000008214460	141.907
37	ZINC000004228267	146.732	77	ZINC000002528486	141.866
38	ZINC000073220104	146.353	78	ZINC000028538573	141.689
39	ZINC000009212426	146.297	79	ZINC000012496598	141.589
40	ZINC000049878197	145.974	80	ZINC000028968107	141.533

### ADME and toxicity prediction

The pharmacological properties of NCB-0846 and 80 candidate compounds were predicted through the ADME module of DS4.5, including aqueous solubility, BBB penetration, CYP2D6 binding, hepatotoxicity, human intestinal absorption, PPB (Table 2). The results showed that all compounds except ZINC000100277550 and ZINC000028538573 were soluble in water (in water at 25°C). Among them, 35 compounds had better water solubility than NCB-0846. In terms of blood-brain barrier permeability, except ZINC000002528486 and NCB-0846 showed Medium permeability, the other compounds were Undefined. CYP2D6 plays an important role in drug metabolism [21]. Except for 13 compounds, all the other compounds including NCB-0846 were predicted to be non-inhibitors of CYP2D6. In addition, we found that NCB-0846 has hepatotoxicity. Among all the candidate compounds, only 34 showed no hepatotoxicity. For human intestinal absorption, NCB-0846 and 5 compounds showed suitable absorption level, while 64 compounds showed poor absorption. Finally, we found that 20 compounds had strong binding ability to plasma protein, while others had weak binding ability.

**Table 2:** ADME (adsorption, distribution, metabolism, excretion) properties of compounds.

**Number**	**Compounds**	**Solubility Level^a^**	**BBB Level^b^**	**CYP2D6^c^**	**Hepatotoxicity^d^**	**Absorption Level^e^**	**PPB Level^f^**
1	ZINC000004099068	3	4	0	0	3	0
2	ZINC000085545908	4	4	0	0	3	0
3	ZINC000049784088	4	4	0	0	3	0
4	ZINC000008552069	4	4	0	1	3	0
5	ZINC000014951658	3	4	0	0	3	0
6	ZINC000013513540	4	4	0	1	3	0
7	ZINC000038143594	3	4	0	0	3	0
8	ZINC000014952116	4	4	0	0	3	0
9	ZINC000040976869	3	4	0	0	2	1
10	ZINC000085826837	2	4	0	0	2	0
11	ZINC000004096878	1	4	0	1	3	1
12	ZINC000004096059	1	4	0	1	3	1
13	ZINC000004096894	2	4	0	1	3	0
14	ZINC000004096684	1	4	0	0	3	1
15	ZINC000085541163	2	4	0	0	2	0
16	ZINC000008551213	4	4	0	1	3	0
17	ZINC000004096889	2	4	0	1	3	0
18	ZINC000009212427	4	4	0	1	3	0
19	ZINC000095562852	3	4	0	0	3	0
20	ZINC000004228266	4	4	0	1	3	0
21	ZINC000004096888	2	4	0	1	3	0
22	ZINC000004096877	1	4	0	1	3	1
23	ZINC000002526388	2	4	1	1	0	1
24	ZINC000042805482	2	4	0	0	2	0
25	ZINC000150338786	1	4	0	1	3	1
26	ZINC000004228235	4	4	0	1	3	0
27	ZINC000004228238	4	4	0	1	3	0
28	ZINC000002005305	4	4	0	1	3	0
29	ZINC000004096893	2	4	0	1	3	0
30	ZINC000014811803	3	4	0	1	3	0
31	ZINC000004228247	4	4	0	1	3	0
32	ZINC000009212425	4	4	0	1	3	0
33	ZINC000004096891	2	4	0	1	3	0
34	ZINC000014951634	3	4	0	0	3	0
35	ZINC000014946303	1	4	0	0	3	0
36	ZINC000002526389	2	4	1	1	0	1
37	ZINC000004228267	4	4	0	1	3	0
38	ZINC000073220104	1	4	0	0	3	1
39	ZINC000009212426	4	4	0	1	3	0
40	ZINC000049878197	1	4	0	0	3	0
41	ZINC000004096890	2	4	0	1	3	0
42	ZINC000014233122	4	4	0	0	3	0
43	ZINC000056897657	1	4	0	1	3	0
44	ZINC000049878225	1	4	0	0	3	0
45	ZINC000085544839	3	4	0	1	3	0
46	ZINC000004097774	2	4	0	0	3	0
47	ZINC000004099069	4	4	0	1	3	0
48	ZINC000014686472	2	4	0	1	3	0
49	ZINC000100288506	1	4	0	1	2	1
50	ZINC000028467879	2	4	0	0	3	0
51	ZINC000038143593	3	4	0	0	3	0
52	ZINC000004095530	1	4	0	1	3	1
53	ZINC000002528509	2	4	1	1	0	1
54	ZINC000011536135	4	4	0	1	3	0
55	ZINC000100590636	3	4	0	0	2	0
56	ZINC000002528510	2	4	1	1	0	1
57	ZINC000014712793	4	4	0	0	3	0
58	ZINC000004228265	4	4	0	1	3	0
59	ZINC000015721425	3	4	0	1	3	0
60	ZINC000004096653	1	4	0	0	3	1
61	ZINC000034944433	2	4	1	0	2	0
62	ZINC000040165309	2	4	0	0	3	0
63	ZINC000100277550	0	4	0	1	3	1
64	ZINC000004228237	4	4	0	1	3	0
65	ZINC000004096892	2	4	0	1	3	0
66	ZINC000002566164	2	4	1	0	3	0
67	ZINC000026671872	1	4	0	1	3	1
68	ZINC000004016719	2	4	1	0	3	0
69	ZINC000003927222	2	4	0	0	3	0
70	ZINC000049872065	3	4	0	0	2	0
71	ZINC000013451339	1	4	1	1	2	1
72	ZINC000017044428	2	4	1	0	3	0
73	ZINC000001530788	3	4	0	1	3	0
74	ZINC000002033589	2	4	1	0	3	0
75	ZINC000004096895	2	4	0	1	3	0
76	ZINC000008214460	3	4	0	0	2	1
77	ZINC000002528486	2	2	1	1	0	1
78	ZINC000028538573	0	4	0	1	2	0
79	ZINC000012496598	2	4	1	0	3	0
80	ZINC000028968107	1	4	1	1	3	1
81	NCB-0846	2	2	0	1	0	1

^a^Aqueous-solubility level: 0 (extremely low); 1 (very low, but possible); 2 (low); 3 (good). ^b^Blood Brain Barrier level: 0 (Very high penetrant); 1 (High); 2 (Medium); 3 (Low); 4 (Undefined). ^c^Cytochrome P450 2D6 level: 0 (Non-inhibitor); 1 (Inhibitor). ^d^Hepatotoxicity: 0 (Nontoxic); 1 (Toxic). ^e^Human-intestinal absorption level: 0 (good); 1 (moderate); 2 (poor); 3 (very poor). ^f^Plasma Protein Binding: 0 (Absorbent weak); 1 (Absorbent strong).

**Figure 1:**
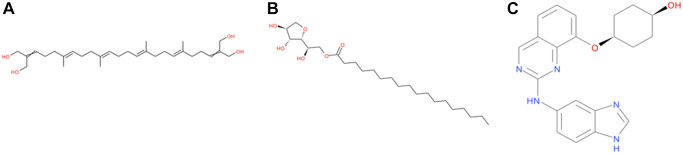
**The structures of NCB-0846 and novel compounds selected from virtual screening.** (**A**) ZINC000040976869; (**B**) ZINC000008214460; (**C**) NCB-0846.

Next, we conducted a comprehensive test and evaluation of the safety of NCB-0846 and the compounds. The rodent carcinogenicity (based on the U.S. National Toxicology Program (NTP) dataset), AMES mutagenicity and developmental toxicity potential (DTP) properties of the candidate compounds were comprehensively texted and evaluated using the TOPKAT module of DS4.5 (Table 3). The results showed that 7 compounds had AMES mutagenicity and 9 compounds had DTP properties. Based on all the above results, ZINC000040976869 and ZINC000008214460 were identified as ideal potential TNIK inhibitors. They have no inhibitory effect on CYP2D6 activity, no hepatotoxicity, and in addition have strong water solubility and plasma protein binding capacity. What's more, they are safe, with almost no rodent carcinogenicity, AMES mutagenicity and DTP. Therefore, we selected them for follow-up studies (Figure 1).

**Table 3:** Toxicities of compounds.

**Number**	**Compounds**	**Mouse NTP^a^**		**Rat NTP^a^**		**AMES^b^**	**DTP^c^**
		**Female**	**Male**	**Female**	**Male**		
1	ZINC000004099068	0.2679	0.0011	0.1309	0.2903	0.0138	0.3672
2	ZINC000085545908	0.2689	0.0216	0.1816	0.4338	0.0001	0.4350
3	ZINC000049784088	0.5414	0.6126	0.2585	0.5119	0.1310	0.5735
4	ZINC000008552069	0.4304	0.3213	0.3737	0.4380	0.2089	0.5275
5	ZINC000014951658	0.1006	0.0005	0.2599	0.4901	0.0001	0.4199
6	ZINC000013513540	0.1048	0.5246	0.0817	0.0774	0.4212	0.6909
7	ZINC000038143594	0.3840	0.4048	0.2651	0.3002	0.1780	0.6137
8	ZINC000014952116	0.0693	0.1092	0.2536	0.4220	0.0027	0.5646
9	ZINC000040976869	0.5233	0.5438	0.3016	0.3061	0.0083	0.5918
10	ZINC000085826837	0.4438	0.3649	0.3071	0.1583	0.0968	0.8155
11	ZINC000004096878	0.5548	0.5323	0.4271	0.5254	0.7150	0.5360
12	ZINC000004096059	0.5280	0.5909	0.4495	0.4886	0.7027	0.5460
13	ZINC000004096894	0.5590	0.4728	0.2861	0.4732	0.4396	0.5218
14	ZINC000004096684	0.5119	0.5523	0.2670	0.1183	0.0040	0.6833
15	ZINC000085541163	0.4438	0.3649	0.3071	0.1583	0.0968	0.8155
16	ZINC000008551213	0.4954	0.4682	0.4326	0.5548	0.2677	0.5404
17	ZINC000004096889	0.5342	0.4427	0.2882	0.4783	0.4573	0.5295
18	ZINC000009212427	0.5163	0.4121	0.4016	0.5459	0.3777	0.5359
19	ZINC000095562852	0.5056	0.7036	0.3098	0.3821	0.0072	0.8049
20	ZINC000004228266	0.4168	0.3199	0.4234	0.4887	0.2981	0.5685
21	ZINC000004096888	0.5342	0.4427	0.2882	0.4783	0.4573	0.5295
22	ZINC000004096877	0.5280	0.5909	0.4495	0.4886	0.7027	0.5460
23	ZINC000002526388	0.2988	0.4387	0.4216	0.4831	0.0000	0.6178
24	ZINC000042805482	0.4438	0.3649	0.3071	0.1583	0.0968	0.8155
25	ZINC000150338786	0.5548	0.5323	0.4271	0.5254	0.7150	0.5360
26	ZINC000004228235	0.4790	0.4253	0.4413	0.5350	0.2979	0.5719
27	ZINC000004228238	0.4971	0.3577	0.4225	0.5350	0.4387	0.5607
28	ZINC000002005305	0.4168	0.3199	0.4234	0.4887	0.2981	0.5685
29	ZINC000004096893	0.5590	0.4728	0.2861	0.4732	0.4396	0.5218
30	ZINC000014811803	0.2888	0.5397	0.2471	0.4883	0.0108	0.7724
31	ZINC000004228247	0.5828	0.3103	0.5004	0.5530	0.2449	0.6479
32	ZINC000009212425	0.5163	0.4121	0.4016	0.5459	0.3777	0.5359
33	ZINC000004096891	0.5342	0.4427	0.2882	0.4783	0.4573	0.5295
34	ZINC000014951634	0.1357	0.0155	0.2285	0.4828	0.0007	0.4616
35	ZINC000014946303	0.4561	0.0888	0.0536	0.0926	0.0005	0.4191
36	ZINC000002526389	0.3270	0.5091	0.4332	0.4770	0.0001	0.6071
37	ZINC000004228267	0.4168	0.3199	0.4234	0.4887	0.2981	0.5685
38	ZINC000073220104	0.3698	0.8174	0.1225	0.1948	0.0000	0.7876
39	ZINC000009212426	0.4997	0.4734	0.4210	0.5459	0.2379	0.5469
40	ZINC000049878197	0.2512	0.0178	0.2279	0.2112	0.0000	0.3970
41	ZINC000004096890	0.5342	0.4427	0.2882	0.4783	0.4573	0.5295
42	ZINC000014233122	0.4109	0.0619	0.0837	0.3412	0.0000	0.4819
43	ZINC000056897657	0.4369	0.0283	0.1680	0.4308	0.0125	0.4668
44	ZINC000049878225	0.2668	0.0250	0.2411	0.2320	0.0000	0.3970
45	ZINC000085544839	0.4514	0.2498	0.3789	0.4380	0.3409	0.5168
46	ZINC000004097774	0.1524	0.6980	0.2119	0.1156	0.3436	0.8829
47	ZINC000004099069	0.4304	0.3213	0.3737	0.4380	0.2089	0.5275
48	ZINC000014686472	0.5430	0.3552	0.2945	0.6421	0.4010	0.9160
49	ZINC000100288506	0.5101	0.5730	0.3726	0.5686	0.7392	0.5505
50	ZINC000028467879	0.0484	0.0005	0.1024	0.4063	0.0000	0.2901
51	ZINC000038143593	0.3840	0.4048	0.2651	0.3002	0.1780	0.6137
52	ZINC000004095530	0.5404	0.5948	0.3783	0.4653	0.6725	0.6038
53	ZINC000002528509	0.2988	0.4387	0.4216	0.4831	0.0000	0.6178
54	ZINC000011536135	0.5652	0.2645	0.4642	0.5358	0.3730	0.6298
55	ZINC000100590636	0.5335	0.5179	0.1415	0.2967	0.0000	0.5242
56	ZINC000002528510	0.3270	0.5091	0.4332	0.4770	0.0001	0.6071
57	ZINC000014712793	0.0466	0.3500	0.0404	0.0599	0.1598	0.7223
58	ZINC000004228265	0.4971	0.4594	0.4469	0.5520	0.2946	0.5461
59	ZINC000015721425	0.3209	0.0261	0.1428	0.2875	0.0552	0.5232
60	ZINC000004096653	0.3678	0.5187	0.1548	0.0622	0.0045	0.6582
61	ZINC000034944433	0.5023	0.4328	0.3268	0.5267	0.0017	0.8358
62	ZINC000040165309	0.3877	0.3185	0.1363	0.3555	0.0000	0.3438
63	ZINC000100277550	0.5285	0.5451	0.3757	0.4925	0.6422	0.5468
64	ZINC000004228237	0.4790	0.4253	0.4413	0.5350	0.2979	0.5719
65	ZINC000004096892	0.5590	0.4728	0.2861	0.4732	0.4396	0.5218
66	ZINC000002566164	0.4697	0.3482	0.3245	0.4859	0.0017	0.8565
67	ZINC000026671872	0.5561	0.5749	0.3484	0.5098	0.6871	0.5830
68	ZINC000004016719	0.4697	0.3482	0.3245	0.4859	0.0017	0.8565
69	ZINC000003927222	0.30837	0.0261	0.1061	0.4246	0.0091	0.4818
70	ZINC000049872065	0.5760	0.6108	0.2151	0.5252	0.0005	0.7927
71	ZINC000013451339	0.2882	0.4219	0.4634	0.5719	0.1479	0.6493
72	ZINC000017044428	0.4697	0.3482	0.3245	0.4859	0.0805	0.8565
73	ZINC000001530788	0.4311	0.4453	0.5081	0.5995	0.2130	0.6364
74	ZINC000002033589	0.4697	0.3482	0.3245	0.4859	0.0017	0.8565
75	ZINC000004096895	0.5590	0.4728	0.2861	0.4732	0.4396	0.5218
76	ZINC000008214460	0.5578	0.6096	0.1421	0.3156	0.0000	0.5522
77	ZINC000002528486	0.2748	0.5642	0.4621	0.4430	0.0001	0.5873
78	ZINC000028538573	0.5501	0.6132	0.4598	0.6757	0.7499	0.6905
79	ZINC000012496598	0.4697	0.3482	0.3245	0.4859	0.0017	0.8565
80	ZINC000028968107	0.1095	0.3210	0.3362	0.0444	0.1148	0.6294
81	NCB-0846	0.6303	0.6125	0.3467	0.3530	0.7322	0.4733

^a^National Toxicology Program (NTP) dataset: <0.3 (Non-Carcinogen); >0.7 (Carcinogen). ^b^AMES mutagenicity :<0.3 (Non-Mutagen); >0.7 (Mutagen). ^c^Developmental Toxicity Potential: <0.3 (Non-Toxic); >0.7 (Toxic).

### Analysis of ligand binding and pharmacophore

**Figure 2:**
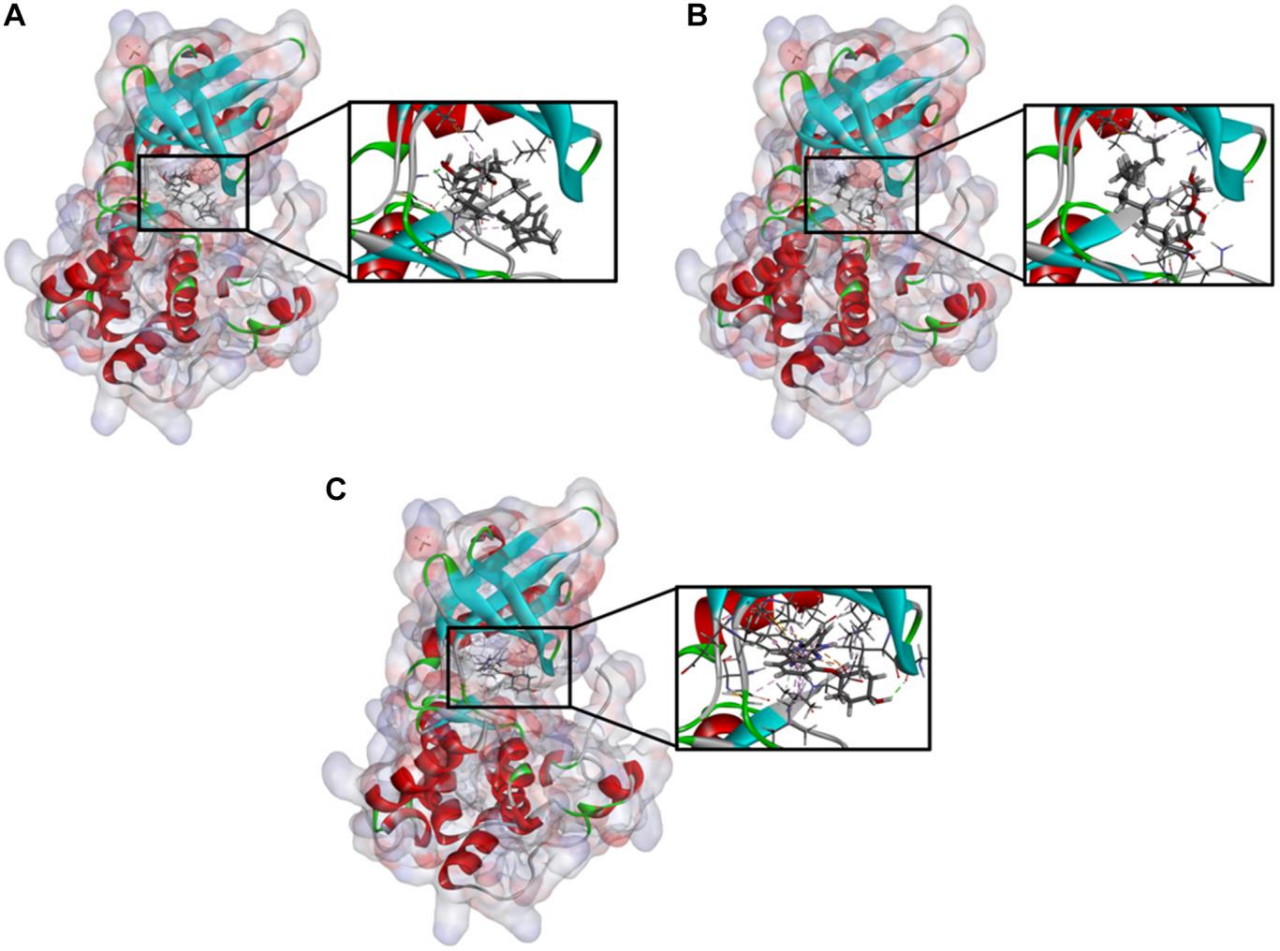
**Schematic drawing of interactions between ligands and TNIK.** The surface of binding areas were added. Blue represents positive charge; red represents negative charge; and ligands were shown in sticks, with the structure around the ligand-receptor junction shown in thinner sticks. (**A**) ZINC000040976869-TNIK complex. (**B**) ZINC000008214460-TNIK complex. (**C**) NCB-0846-TNIK complex.

**Figure 3:**
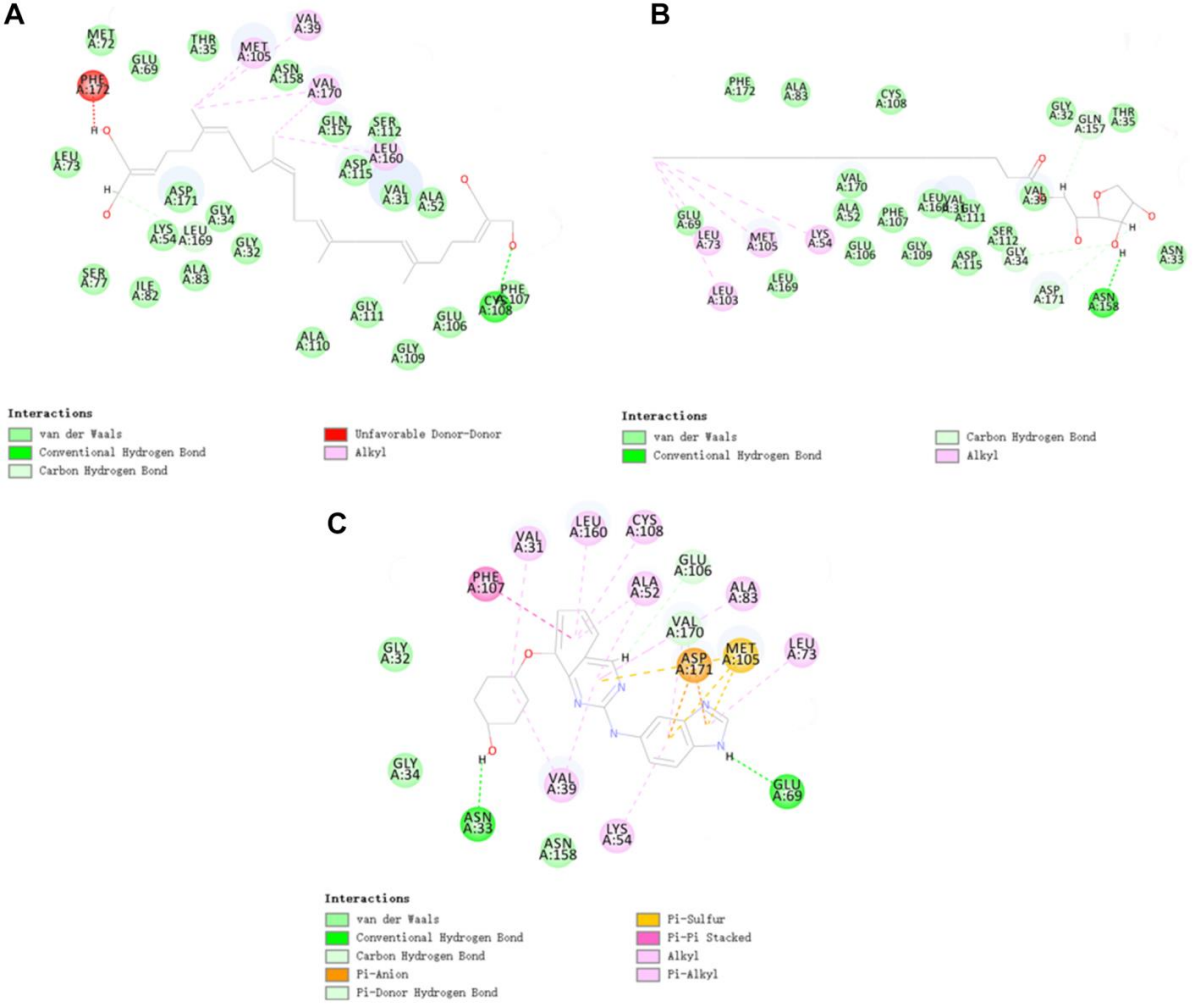
The inter-molecular interaction of the predicted binding modes of (**A**) ZINC000040976869 to TNIK; (**B**) ZINC000008214460 to TNIK and (**C**) NCB-0846 to TNIK.

**Figure 4:**
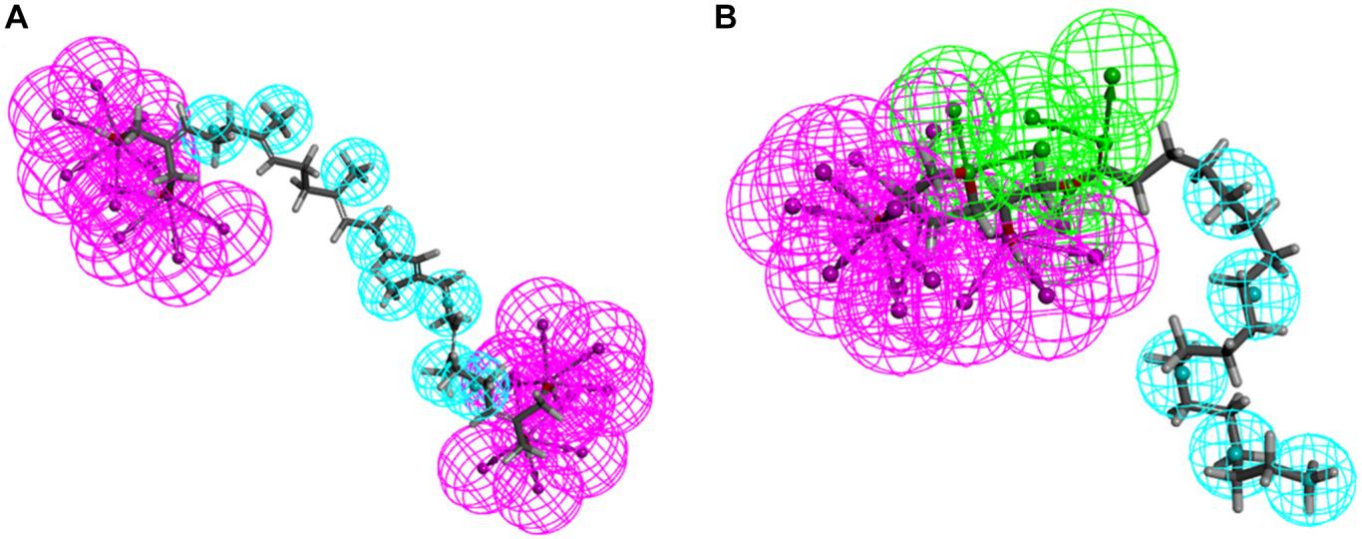
**Pharmacophore predictions using 3D-QSAR.** (**A**) ZINC000040976869: Blue represents hydrophobic center and purple represents hydrogen donor. (**B**) ZINC000004096987: Green represents hydrogen acceptor, blue represents hydrophobic center and purple represents hydrogen donor.

In order to further analyze the binding mechanism of ligands and TNIK, we used the CDOCKER module of DS4.5 for calculation [22]. The RMSD between the crystal structure and the docking posture was 0.6Å, which indicates that the use of CDOCKER module was reliable. After applying the force field of CHARMm36, we connected the two compounds and NCB-0846 into the 3D structure of TNIK to calculate the interaction energy of CDO CKER. Results as shown in Table 4, the interaction energy of ZINC000040976869 was −57.066 Kcal/mol and the interaction energy of ZINC000008214460 was −58.181 Kcal/mol, which was significantly lower than that of NCB-0846 (−52.062 Kcal/mol). This indicated that both compounds may bind to TNIK more easily and stably compared to NCB-0846. Then, we performed structural analysis of the interactions between TNIK and ligands, including hydrogen bonds and other types of hydrophobic bonds (Figures 2, 3, Tables 5, 6). There were two hydrogen bonds formed by ZINC000040976869 and TNIK (A:CYS108:HN-ZINC000040976869:O24, ZINC000040976869:H80-A:LEU169:O). In addition, five Alkyl interactions were formed in this complex. Similarly, four hydrogen bonds were formed by ZINC000008214460 and TNIK (ZINC000008214460:H76-A:ASN158:OD1, A:GLY34: HA1-ZINC000008214460:O30, ZINC000008214460: H67-A:GLN157:O, ZINC000008214460:H75-A:ASP171:OD2) and four Alkyl interactions were formed. In addition, the calculation results of pharmacodynamic groups of the two compounds were shown in Figure 4. ZINC000040976869 had 29 feature pharmacophores, including hydrogen donors and hydrophobic centers. ZINC000008214460 had 31 feature pharmacophores, including hydrogen bond acceptors, hydrogen donors and hydrophobic centers.

**Table 4:** CDOCKER interaction energy of compounds with TNIK.

**Compounds**	**CDOCKER interaction energy (Kcal/mol)**
ZINC000040976869	−57.066
ZINC000008214460	−58.181
NCB-0846	−52.062

**Table 5:** Hydrogen bond interaction parameters for each compound and TNIK residues.

**Receptor**	**Compound**	**Donor atom**	**Receptor atom**	**Distances (Å)**
TNIK	ZINC000040976869	A:CYS108:HN	ZINC000040976869:O24	1.92
		ZINC000040976869:H80	A:LEU169:O	2.97
	ZINC000008214460	ZINC000008214460:H76	A:ASN158:OD1	2.38
		A:GLY34:HA1	ZINC000008214460:O30	2.55
		ZINC000008214460:H67	A:GLN157:O	2.49
		ZINC000008214460:H75	A:ASP171:OD2	2.76
	NCB-0846	58C:H48	A:GLU69:OE2	2.28
		58C:H49	A:ASN33:O	2.06
		A:VAL170:HA	58C:N27	2.42
		58C:H29	A:GLU106:O	2.77
		A:ASP171:HN	58C	2.19

**Table 6:** Pi-Anion interaction, Pi-Sulfur interaction, Pi-Pi Stacked interaction, Pi-Alkyl interaction and Alkyl interaction parameters for each compound and TNIK residues.

**Receptor**	**Interaction parameters**	**Compound**	**Donor atom**	**Receptor atom**	**Distances (Å)**
TNIK	Pi-Anion interaction	NCB-0846	A:ASP171:OD1	NCB-0846	4.15
			A:ASP171:OD1	NCB-0846	3.23
	Pi-Sulfur interaction	NCB-0846	A:MET105:SD	NCB-0846	5.28
			A:MET105:SD	NCB-0846	3.97
			A:MET105:SD	NCB-0846	3.69
	Pi-Pi Stacked interaction	NCB-0846	A:PHE107	NCB-0846	5.62
	Pi-Alkyl interaction	NCB-0846	NCB-0846	A:VAL39	4.92
			NCB-0846	A:ALA52	3.71
			NCB-0846	A:ALA52	3.7
			NCB-0846	A:ALA83	5.5
			NCB-0846	A:CYS108	4.87
			NCB-0846	A:LEU160	4.68
			NCB-0846	A:VAL170	4.51
			NCB-0846	A:LYS54	5.04
			NCB-0846	A:LEU73	4.57
			NCB-0846	A:VAL170	5.13
			NCB-0846	A:VAL170	5.03
	Alkyl interaction	ZINC000040976869	ZINC000040976869:C1	A:VAL39	4.41
			ZINC000040976869:C1	A:MET105	5.31
			ZINC000040976869:C1	A:VAL170	4.15
			ZINC000040976869:C7	A:LEU160	5.25
			ZINC000040976869:C7	A:VAL170	5.24
		ZINC000008214460	ZINC000008214460:C1	A:LYS54	4.94
			ZINC000008214460:C1	A:LEU73	4.33
			ZINC000008214460:C1	A:LEU103	4.87
			ZINC000008214460:C1	A:MET105	4.36
		NCB-0846	A:VAL31	NCB-0846	5.06
			A:VAL39	NCB-0846	4.12

### Molecular dynamics simulation

**Figure 5:**
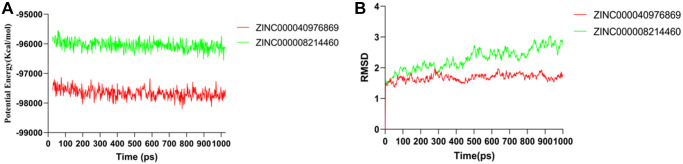
**Results of molecular dynamics simulation of two complexes.** (**A**) Potential energy; (**B**) Average backbone RMSD.

**Figure 6:**
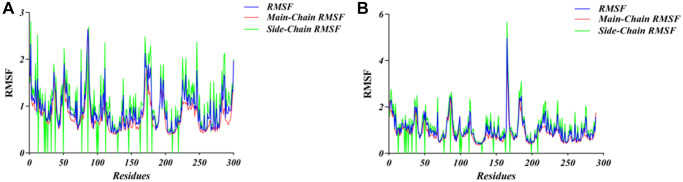
**RMSF trajectories of the system during molecular dynamics simulation.** (**A**) ZINC000040976869-TNIK complex. (**B**) ZINC000008214460-TNIK complex.

The complex initial conformation of TNIK-compounds was obtained by molecular docking module of CDOCKER. Then, the stability of the complex was simulated by molecular dynamics using the Standard Dynamics Cascade module of DS4.5 under dynamic conditions. Under the action of CHARMm force field, the motion of molecules was displayed dynamically. The potential energy and RMSD results obtained after 1ns simulation are shown in Figure 5. We can see from the figure that the RMSD trajectorie of ZINC000040976869 reaches equilibrium after 300ps, while ZINC000008214460 reaches equilibrium after 800ps. And over time, their potential energy and RMSD tend to be stable. In addition, in order to analyze the volatility of various amino acids in the complex during molecular dynamics simulation, we calculated the RMSF values of all amino acids during simulation. It can be seen from Figure 6 that the TNIK-ZINC000040976869 complex fluctuated greatly around the amino acids Glu12, Gly96 and Arg180, while the TNIK-ZINC000008214460 complex fluctuated greatly around the amino acids Ile13, Gly96, Asn186 and Pro206.

## DISCUSSION

As a major cancer, the incidence of CRC has been increasing in recent years, accounting for 10% of cancer-related mortality [1]. This undoubtedly places a heavy health and economic burden on the world every year. Although various treatment methods have made great progress, the cure rate of CRC is still not optimistic. At present, the widely used CRC targeting drugs are mainly targeted at VEGF (bevacizumab) and EGFR (cetuximab and panitumab). But this survival benefit is also limited to a few months [23]. Therefore, it is of great significance to find new target drugs in the molecular signaling pathway of CRC. It has been demonstrated that 90% of CRC patients have typical Wnt/β-catenin signaling pathway gene mutations [9]. Activation of the Wnt signaling pathway leads to the generation of cancer stem cells [9]. The mutation of APC tumor suppressor gene is more than 80% in CRC and is the earliest step in the carcinogenesis of CRC [24]. Above all, it is perspective to search for molecules downstream of APC in the Wnt/β-catenin signaling pathway as targets for drug therapy.

As a member of serine/threonine protein kinase family, TNIK has been proved to play a cancer-promoting role in CRC, gastric cancer, lung cancer, prostate cancer and other diseases [25–27]. As the most downstream molecule of the Wnt/β-catenin signaling pathway, its feasibility as a drug target has been confirmed by many researches [7, 14]. In CRC, inactivated APC results in β-catenin accumulation that cannot be degraded. The excess β-catenin will be transported into the nucleus and form a transcription complex with TCF4. As an activating kinase of the β-catenin/TCF4 transcriptional complex, TNIK phosphorylates the TCF4 protein at the conserved serine 154 residue, thereby activating the transcriptional complex. This will fully activate Wnt signaling pathway, thus promoting the growth and invasion of tumor. The study of Gui et al. demonstrated that TNIK gene knockout can block the activation of Wnt signaling and inhibit the growth of tumor cells [28]. At present, several TNIK inhibitors have been developed, and the most representative of which is NCB-0846. However, due to the poor pharmacological characteristics and low activity, it has not been used in clinic so far. As a practical TNIK inhibitor, NCB-0846 can bind to the ATP active pocket of TNIK, making it unable to phosphorylate TCF4, thereby exerting a tumor suppressive effect [7, 29]. Consequently, we used NCB-0846 as a reference drug to screen for more ideal TNIK inhibitors.

To explore effective TNIK inhibitors, 14962 natural, named and purchasable small molecules were obtained from the ZINC15 database for screening. Based on the binding stability, pharmacological properties, toxicity and stability of compounds, the ideal TNIK potential inhibitors were determined. LibDock is a preliminary screening method for small molecules, the higher the score, the more stable the receptor-ligand conformation, and the more optimized the energy. According to the results of LibDock, we found that 1246 compounds scored higher than the reference drug NCB-0846. Thus, these compounds may form more stable complexes with TNIK compared to NCB-0846. The top 80 natural compounds were selected based on LibDock score for further analysis.

Next, we analyzed the pharmacological and toxicological properties of the candidate compounds and NCB-0846 using ADME and TOPKAT modules. Among them, ZINC000040976869 and ZINC000008214460 attracted our attention. First of all, these two compounds have strong binding ability to plasma proteins, good water solubility, can be absorbed by the intestinal tract and can not pass through the blood-brain barrier. In addition, they have no inhibitory effect on CYP2D6 and no hepatotoxicity. On the other hand, the rodent carcinogenicity, AMES mutagenicity and developmental toxicity potential of these two compounds are relatively low. Compared with NCB-0846, these two compounds have less hepatotoxicity, better water solubility, do not cross the blood-brain barrier and have lower toxicity. Based on the above results, it shows that ZINC000040976869 and ZINC000008214460 have good prospects in drug development and can be used as ideal lead compounds for further analysis. Although other candidate compounds have not been selected, they can reduce toxicity and improve pharmacological properties by adding or modifying the groups they contain. For this reason, they can still be listed as candidate drugs.

CDOCKER is a technology that produces high-precision molecular docking results in the CHARMm position, which is more precise than LibDock. CDOCKER interaction energy is an index to evaluate the affinity of receptor-ligand. The lower it is, the higher the affinity between ligand and receptor. Our results showed that the CDOCKER interaction energy of ZINC000040976869 and ZINC000008214460 were −57.066 Kcal/mol and −58.181 Kcal/mol respectively, which was lower than that of NCB-0846 (−52.062 Kcal/mol). This indicates that the affinity and stability of the two small molecules we selected were higher than those of NCB-0846. In addition, the protein binding sites of NCB-0846 and these two compounds are the same, and they all have axisymmetric structures. To sum up, compared with NCB-0846, they may have better inhibitory effect and higher safety.

In the calculation of the pharmacophore of the compound, we found that ZINC000040976869 displayed several hydrogen donors and hydrophobic centers, with a total of 29 pharmacophores. Similarly, ZINC000008214460 displayed several hydrogen bond acceptors, hydrogen donors and hydrophobic centers, with a total of 31 pharmacodynamic groups. This means that in future research, the two compounds have the potential to add different functional groups to improve drugs and improve anti-cancer efficacy.

Next, we used molecular dynamics simulations to analyze the two optimal conformations of TNIK-compounds complexes. This can scientifically evaluate its stability in the natural environment. We found that RMSD trajectories and interaction energy of ZINC000040976869 and ZINC000008214460 tended to be stable after 300ps and 800ps, respectively. This indicated that the hydrogen bonds and Alkyl interactions between the two compounds and TNIK played a stable role in the formation of the complexes. Complexes can exist stably in natural environment. In addition, by analyzing the RMSF trajectories of the two compounds, we can see that TNIK- ZINC000040976869 complex fluctuates greatly around amino acids Glu12, Gly96 and Arg180, while TNIK- ZINC000008214460 complex fluctuates greatly around amino acids Ile13, Gly96, Asn186 and Pro206. The fluctuation trends of RMSF trajectories of the two complexes are different, which means that the fluctuations of amino acids of different complexes are different in the simulation process. Therefore, the amino acid positions with large fluctuations in RMSF of these two compounds can be optimized in the subsequent drug modification process.

The most important step in drug design is to identify reasonable lead compounds, followed by continuous modification and improvement to make them clinically applicable. Based on the above studies, the two natural compounds we have identified can provide great help for the development of targeted drugs for CRC. Although our study was rigorously designed, there are some limitations. For example, we may need to conduct animal experiments, molecular biology experiments, etc. to further confirm our results. In the future research, we can also evaluate LD50, ED50, Maximum Tolerated Dosage (MTD) and Aerobic Biodegradability (A.B.) and other indicators.

## CONCLUSION

In this study, a series of biological and chemical methods (Virtual Screening, Molecule Docking, ADME, Toxicity Prediction, Molecular Dynamics Simulation) were used under computer assistance to screen and analyze the potential TNIK inhibitors. After comprehensive analysis, two natural fractions, ZINC000040976869 and ZINC000008214460, were identified as ideal drug candidates. They have strong affinity with TNIK and good pharmacological properties and safety, which makes them promising to be further developed into new CRC targeted drugs.

## MATERIALS AND METHODS

### Docking software and ligand library

Discovery Studio 4.5 (DS4.5, Accelrys, Inc.) is a software for molecular modeling and environmental simulation in the field of life sciences. It has various functions including characterization of protein, three-dimensional molecular construction, drug design, molecular docking, database screening and so on. Through this method, many lead compounds and drug candidates have been identified and improved. Our candidate small molecules were obtained from natural products (NP) database in ZINC15 database. ZINC15 is a free database of commercial compounds provided by Irwin and Shoichet laboratories in the Department of Pharmaceutical Chemistry of UCSF (University of California, San Francisco, USA). First of all, we used the LibDock module of DS4.5 to screen the candidate compounds. Furthermore, ADME (absorption, distribution, metabolism, excretion) and TOPKAT (toxicity prediction by computer assisted technology) modules of the software were used to analyze the pharmacological characteristics of compounds. Then, the CDOCKER module was used to dock the compounds with TNIK more accurately. Finally, in order to analyze the stability of TNIK-ligand complexes, molecular dynamics simulation was carried out.

### Virtual screening using LibDock

**Figure 7:**
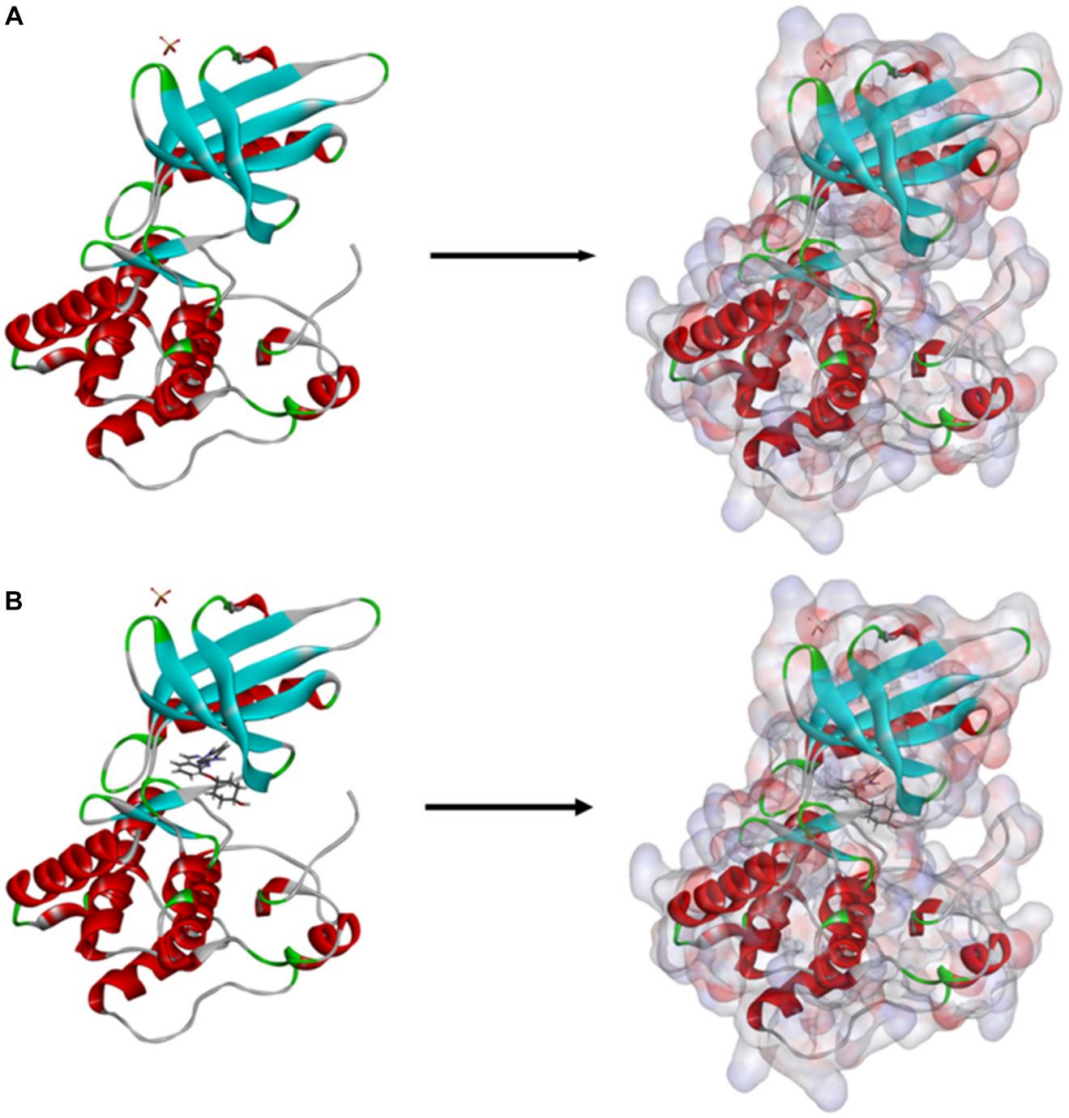
(**A**) The molecular structure of TNIK. Initial molecular structure was shown, and the surface of the molecule was added. (**B**) The complex structure of TNIK with NCB-0846. Initial complex structure was shown, and the surface of the complex was added. Blue represented positive charge, red represented negative charge.

Firstly, we obtained the crystal structure of TNIK in complex with NCB-0846 from the protein database (PDB) (PBD ID: 5d7a, 2.9 Å). LibDock is a rigid docking program in DS4.5. It used a grid located at the binding position and uses polar and apolar probes to calculate the hotspots of the complex. Then hot spots were used to coordinate ligands for favorable binding. The 3D chemical structure of TNIK was shown in Figure 7. The structure was prepared by removing crystal water, ligands and other heteroatoms, then adding hydrogen, protonation, ionization and energy minimization. Furthermore, Smart Minimiser algorithm and CHARMm force field (Cambridge, MA, USA) were used to minimize energy [30]. In order to screen TNIK inhibitors more accurately, the binding pocket region of TNIK and NCB-0846 was selected as the docking site. 9.3 Å was set as the spherical docking site diameter based on the PDB site records. Next, all prepared small molecules were butted to the defined active sites for virtual screening using LibDock. Finally, the compounds with different docked poses were ranked according to LibDock score.

### ADME and toxicity prediction

The ADME (Absorption, Distribution, Metabolism and Excretion) module of DS4.5 was used to evaluate the water solubility, blood-brain barrier (BBB) permeability, cytochrome P450 2D6 (CYP2D6) inhibition, hepatotoxicity, human-intestinal absorption level and plasma protein binding (PPB) level of the selected compounds. Additionally, the TOPKAT (Toxicity Prediction by Komputer Assisted Technology) module was employed to assess and analyze the toxicity of all potential compounds. This module can accurately analyze and verify the toxicity and environmental effects of compounds based on their 2D molecular structures. The molecular toxicities we have mainly analyzed including rodent carcinogenicity, AMES mutagenicity, developmental toxicity potential (DTP). We comprehensively analyzed the pharmacological properties predicted by ADME and the results of toxicity analysis to select potential candidate TNIK inhibitors.

### More precise molecular docking and pharmacophore prediction

The CDOCKER module of DS4.5 was used to conduct molecular docking research based on CHARMm36 force field. During the docking process, the receptor is held rigid, while the ligands are allowed to flex, so that higher precision docking results can be obtained. Initially, we obtained the crystal structure of TNIK from PBD. Then, DS4.5 was employed to prepare and process protein crystals. Considering that fixed water molecules may affect the formation of receptor-ligand complexes during rigid and semi-flexible docking, we removed crystalline water molecules from protein before this process [31, 32]. After that, hydrogen atoms were added to protein. In order to make the docking results more reliable, we removed NCB-0846 from the protein structure, and then re-docked into the crystal structure of TNIK. The binding site of TNIK was defined as a binding sphere with a diameter of 9.3Å centered on the NCB-0846 binding region. CHARMm36 force field is applied to the docking process of receptor and ligands. During the docking process, the ligand gradually recognized and bound with the residues in the receptor binding sphere. The CHARMm energy (interaction energy plus ligand strain) based on each complex posture and the interaction energy representing ligand binding affinity were calculated after docking process. The ligand poses with the lowest interaction energy and appropriate docking direction was selected for follow-up research. In addition, the 3D-QSAR pharmacophore generation module was used to display the pharmacophore of the compound. Up to 255 conformations were generated per molecule to represent a small molecule. However, only conformations with the energy below 10 kcal/mol were preserved.

### Molecular dynamics simulation

To further investigate the binding process between TNIK and the two candidate compounds, molecular dynamics simulations were performed on the two optimal ligand-receptor complexes. We employed the Solvation module of DS4.5 to put the ligand-receptor complex into an orthogonal box and solvated it with the explicit periodic boundary solvent-water model. In order to simulate the action in physiological environment, chlorides with ionic strength of 0.145 were added to the system. After that, we applied CHARMm force field to the system and minimized the energy (500 steps for steep descent and 500 steps for conjugate gradient). The temperature of the system was slowly driven from 50K to 300K, and the equilibrium simulation time was 20 ps. Molecular dynamics simulations (production module) were run for 1 ns with time step of 2 ps. This process was carried out with NTP (Atmospheric Pressure and Temperature) system at a constant temperature of 300K. The particle mesh Ewald (PME) algorithm was used for long-range electrostatic calculations, and the Linear Constraint Solver (LINCS) algorithm was used to identify all hydrogen-containing bonds. In the next process, we used DS4.5 to analyze molecular dynamics trajectories, including Root-Mean-Square Deviation (RMSD), Root-Mean-Square Fluctuation (RMSF), potential energy and structural characteristics.
